# Effect of donor GSTM3 rs7483 genetic variant on tacrolimus elimination in the early period after liver transplantation

**DOI:** 10.7717/peerj.18360

**Published:** 2024-10-23

**Authors:** Tao Zhang, Xiaorong Chen, Yuan Liu, Lei Zhang

**Affiliations:** 1 Department of Organ Transplantation, The Third Affiliated Hospital of Sun Yat-sen University, Guangzhou, China; 2 Department of General Surgery, Shanghai General Hospital, Shanghai Jiao Tong University School of Medicine, Shanghai, China

**Keywords:** Glutathione S-transferase mu, CYP3A5, Genetic variant, Tacrolimuspharmacokinetics, Liver transplantation

## Abstract

**Purpose:**

Glutathione S-transferase mu (GSTM) belongs to the group of phase II drug-metabolizing enzymes, and the GSTM1 genetic variant has been reported to have a potential association with the metabolism of immunosuppressive drug after renal transplantation. The effect of donor and recipient GSTMs genetic variants on tacrolimus (Tac) metabolism was the focus of our investigation in this study.

**Methods:**

A total of 203 liver transplant patients were recruited for the study. In the training set (*n* = 110), twenty-one SNPs in five genes (GSTM1-5) were genotyped by the drug-metabolizing enzymes and transporter (DMET) microarray. CYP3A5 rs776746 and GSTM3 rs7483 were genotyped using a Mass ARRAY platform in the validating set (*n* = 93).

**Results:**

Tac C/D ratios of donor GSTM3 rs7483 AA carriers were significantly lower than those with the G allele at weeks 1, 2, 3 and 4 after liver transplantation (LT). Multivariate analysis was conducted on the training set and validating set, donor and recipient CYP3A5 rs776746, donor GSTM3 rs7483 and total bilirubin were identified as independent predictors of Tac C/D ratios in the early period after LT. Combining CYP3A5 rs776746 and donor GSTM3 rs7483 genotypes, Tac C/D ratios were observed to be increasingly lower with increasing numbers of alleles associated with fast metabolism. Moreover, the risk of a supratherapeutic C_0_ (Tac > 15 ug/L) was significantly higher for poor metabolizers than the other groups at week 1 after LT.

**Conclusions:**

There was a significant association between the donor GSTM3 rs7483 genetic variant and Tac metabolism in the early period after LT. Genotype classification might have a better predictive ability of the initial Tac doses.

## Introduction

Tacrolimus (Tac) is a pivotal immunosuppressive agent for the prevention of allograft rejection in liver transplantation (LT). Posttransplant survival has greatly improved with its use. However, the clinical use of Tac is complicated due to its narrow therapeutic index and the large inter- and intra-individual variation in its pharmacokinetics  (Virunya et al., 2024; Siqi et al., 2024). Underdosing of Tac may cause underimmunosuppression and acute graft rejection, whereas overdosing puts patients at the risk of serious post-transplant complications, including infection, nephrotoxicity, neurotoxicity and diabetes mellitus (Oren et al., 2024; Azhie et al., 2021; Pageaux et al., 2009). Therefore, there has been increasing recognition of the need for means of individualizing Tac treatment to rapidly achieve target blood concentration in the early period after LT.

Pharmacogenetics has the potential to explain 20%–95% of the inter- and intra-individual variation observed in drug metabolism and treatment response (Evans & McLeod, 2003). Individual single nucleotide polymorphisms (SNPs) may change the expression or biologic activity of protein that has a physiological effect on the organism (Siqi et al., 2024). Tac is a substrate of metabolic and transport enzymes, the genetic variants of cytochrome P-450 3A4/5 (CYP3A4/5), multidrug resistance protein 1 (ABCB1) could partly alter the metabolism and clearance of Tac in organ transplantation (Liu et al., 2022; Aouam et al., 2015; Liu et al., 2013). However, the inter- and intra-individual variation of Tac pharmacokinetics could not be fully explained by these SNPs, and additional determinants remain to be uncovered.

Glutathione S-transferase mu (GSTM) are some of the most abundant GSTs found in the human liver and brain (Uno et al., 2020), and belong to the group of phase II drug-metabolising enzymes that facilitate the detoxification of toxic chemical, therapeutic drugs and products of oxidative stress (Doerksen et al., 2023; Uno et al., 2020). There are five distinct human isoforms in the GSTM cluster (GSTM1, GSTM2, GSTM3, GSTM4, GSTM5). In addition, a number of polymorphisms have been reported and characterized in these genes (Santos et al., 2019; Rodríguez et al., 2018; Tatemichi et al., 2009). The genetic variants of these GSTMs have the potential to alter an individual’s susceptibility to carcinogens and toxins, and influence the toxicity and efficacy of drug treatment. And a previous study has reported that the polymorphisms of GSTM1 had a potential association with the elimination of Tac in renal transplant recipients (Hayes & Strange, 2000; Singh et al., 2009; Kearns et al., 2002).

The current study was based on our drug-metabolizing enzymes and transporter (DMET) microarray, which contains 1936 genetic variants in 225 related genes. Firstly, we investigated the association between GSTMs SNPs within DEMT microarray and Tac pharmacokinetics from our study subjects, the donors and recipients of 110 liver transplantations (Cohort A). These genetic variants included GSTM2 (rs530021), GSTM3 (rs7483, rs4646412), GSTM4 (rs506008), GSTM5 (rs1296954, rs11807). The significant markers were then validated in the test set (Cohort B: *n* = 93).

## Patients & Methods

### Patients

From Jan 2017 and Dec 2020, we meticulously screened patients who underwent orthotopic liver transplantation at The Third Affiliated Hospital of Sun Yat-sen University, China (Cohort A = 110, Cohort *B* = 93.) The criteria were: (1) age ≥18 years, (2) LT from DCD/DBD, (3) immunosuppressive regimen was triple therapy incorporating tacrolimus, mycophenolate, and steroid, (4) Signed informed consent. The exclusion criteria were: (1) multiorgan transplant patients, (2) follow-up time less than 1 month, (3) Immunosuppressive regimen altered (*e.g.*, to cyclosporin). The patient characteristics were summarized in Table 1.

**Table 1:** Baseline demographic characteristics.

	Total population (*n* = 203)	
Demographic variables	Cohort A (*n* = 110)	Cohort B (*n* = 93)
Recipient age (Years)	47.48 ± 9.20	48.85 ± 10.86
Recipient gender (male/female, n)		
Child-pugh score	91 (82.7%) /19 (17.3%)	74 (79.6%)/19 (20.4%)
Primary disease (n)	7.12 ± 2.12	9.38 ± 2.44
HBV cirrhosis		
HCV cirrhosis		
Any with HCC	29 (26.4%)	58 (62.3%)
Autoimmune cirrhosis	–	1 (1.1%)
Alcoholic cirrhosis	69 (62.7%)	25 (26.9%)
Primary biliary cirrhosis	4 (3.6%)	1 (1.1%)
Wilson Disease	2 (1.8%)	1 (1.1%)
Schistosomal cirrhosis	3 (2.7%)	4 (4.3%)
Budd-Chiari syndrome	2 (1.8%)	2 (2.1%)
	1 (1.0%)	–
	–	1 (1.1%)

### Ethics statement

Human participants written informed consent was obtained from the subjects or the next-of-kin. The retrospective research was approved by the Ethics Committee of The Third Affiliated Hospital of Sun Yat-sen University (A2023-232-01). All procedures were performed in accordance with the 1964 Declaration of Helsinki and its later amendments or comparable ethical standards. Assurances were made to ensure that no livers were obtained from the executed prisoners (1) Voluntary organ donation (DCD/DBD), (2) human participants written informed consent was obtained from the next-of-kin of donors, (3) the quality of donor’s liver meets the transplantation standard, (4) China made posthumous voluntary donation the only legitimate source of organs in 2015).

### Data collection

Therapeutic drug monitoring (TDM) was performed routinely after LT. The C_0_ was measured in laboratories by the Pro-TracTMII tacrolimus ELISA kit (Diasorin) with a microparticle enzyme immunoassay (ELx 800NB analyzer; BioTek), using the whole blood collected before the morning administration. We used the Tac C/D ratio (ug/L per mg/kg) as a measure of Tac pharmacokinetics, calculating it by dividing the trough concentration (ug/L) by the dosage adjusted for body weight (mg/kg). Clinical data encompassed demographic insights (age, gender, child-pugh score, primary liver disease), liver function index (ALT, AST, TB, DB, Alb), and renal function index (Cr, Urea).

### Genomic DNA isolation & genotyping

Genomic DNA obtained from donor and recipient hepatic samples was purified using the AllPrep DNA/RNA mini-kit (Qiagen, Hilden, Germany), previously secured at −80 °C. In the training set (Cohort A), genomic DNA from 110 patients was genotyped by Affymetrix DMET Plus array according to the molecular inversion probe (MIP) technology as previously described (Dumaual et al., 2007; Di Martino et al., 2011a; Di Martino et al., 2011b). And in the validated set (Cohort B), genotyping of CYP3A5 rs776746 and GSTM3 rs7483 was conducted using the Sequenom MassARRAY SNP genotyping platform (Sequenom, San Diego, CA, USA) (Gabriel, Ziaugra & Tabbaa, 2009). The sequencing primers for rs776746 and rs7483 were as follows: rs776746: forward 5′-AGGAAGCCAGACTTTGATCATTATGTT-3′, reverse 5′-GAGAGTGGCATAGGAGATACCCA-3′; rs7483: forward 5′-CCAGTATCGCAGCGATTC AATT-3′, reverse 5′-GCCTACTTACAGTCTGATCAGTTCTG-3′.

### Statistical analysis

SPSS version 19.0 (SPSS, Chicago, IL, USA) was used for statistical analysis. Genetic equilibrium and allele distribution were analyzed using the SHEsis software platform (Shi & He, 2005). Tac C/D ratios were assessed for normality of distribution and logarithmically transformed if non-normal. Mean substitution where we substituted the missing values. Tac C/D ratios between genotype groups were analyzed using Student independent *t*-tests or Mann–Whitney test. Given the null hypothesis that the group means are equal, the ANOVAs were conducted. We utilized stepwise multiple linear regression to evaluate the impact of the CYP3A5 rs776746 and GSTM3 rs7483 genetic variant on Tac C/D ratios, along with clinical characteristics such as ALT, AST, TB, DB, Alb, Cr, Urea. Variables with a univariate *p* < 0.10 were included in multivariate analysis. The enter method was used to confirm the results derived from the training dataset. We assessed the impact of genotype cluster on the risk of Tac blood concentrations >15 ug/L using logistic regression analysis. Statistical significance was determined by two-tailed *p*-values of less than 0.05.

## Results

### Gene distribution

The distribution of genotypes of CYP3A5 rs776746 and GSTM1-5 SNPs within the DEMT microarray is presented in Table S1. Fourteen SNPs with no variants in our study population were excluded for further analysis. The remaining seven SNPs conformed to Hardy–Weinberg equilibrium (*P* > 0.05). No significant linkage disequilibrium was observed between the CYP3A5 rs776746 and the six GSTM2-5 SNPs.

### Influence of CYP3A5 and GSTM2-5 genetic vatiants on Tac elimination (Cohort A)

The association between donor CYP3A5 and GSTM2-5 SNPs and Tac C/D ratios in the early period after LT was shown in Table 2. Among CYP3A5 rs776746 carriers, those with AA/AG genotypes have been observed to have lower Tac C/D ratios than GG genotype carriers at weeks 1, 2, 3, 4 (*p* = 0.005, 0.007, 0.002, <0.001, respectively). Tac C/D ratios of donor GSTM3 rs7483 AA genotype were 231.0 ± 164.9, 127.3 ± 73.6, 120.4 ± 82.4 and 116.1 ± 71.1 at weeks 1, 2, 3 and 4 respectively. For AG and GG genotype carriers, the corresponding Tac C/D ratios at each time point were 328.2 ± 243.6, 195.1 ± 146.6, 213.4 ± 219.6 and 235.20 ± 180.3. The differences were significant (*p* = 0.035, 0.010, 0.035, 0.002, respectively).

**Table 2:** Tac C/D ratios according to donor CYP3A5 and GSTMs genotypes after drug initiation (Cohort A, *n* = 110).

			Week 1		Week 2		Week 3		Week 4	
Gene	SNP	Genotype	C/D ratios	P	C/D ratios	P	C/D ratios	P	C/D ratios	P
CYP3A5	rs776746	AA+AG	215.0 ± 140.3	0.005	139.1 ± 97.0	0.007	129.6 ± 120.3	0.002	114.7 ± 82.9	<0.001
		GG	348.9 ± 253.2		180.8 ± 135.2		203.0 ± 203.0		229.4 ± 273.4	
GSTM2	rs530021	CC	265.0 ± 189.3	0.739	159.2 ± 116.5	0.498	168.0 ± 171.9	0.464	176.5 ± 218.9	0.256
		CG+GG	341.9 ± 301.4		157.4 ± 130.6		142.3 ± 144.5		135.0 ± 111.5	
GSTM3	rs7483	AA	231.0 ± 164.9	0.035	127.3 ± 73.6	0.010	120.4 ± 82.4	0.035	116.1 ± 71.1	0.002
		AG+GG	328.2 ± 243.6		195.1 ± 146.6		213.4 ± 219.6		235.20 ± 180.3	
	rs4646412	GG	277.7 ± 215.8	0.822	154.5 ± 115.8	0.096	162.8 ± 167.1	0.522	171.0 ± 214.1	0.174
		GT+TT	267.8 ± 151.0		213.3 ± 149.3		176.3 ± 181.0		163.2 ± 85.7	
GSTM4	rs506008	GG	263.8 ± 193.7	0.363	157.4 ± 116.3	0.946	165.1 ± 170.0	0.868	173.1 ± 216.2	0.652
		GA+AA	367.4 ± 297.2		170.0 ± 134.7		156.2 ± 155.1		150.2 ± 117.2	
GSTM5	rs1296954	GG	272.8 ± 189.2	0.764	155.4 ± 116.2	0.720	144.2 ± 146.3	0.149	130.8 ± 82.4	0.089
		AG+AA	284.3 ± 248.3		165.7 ± 123.0		202.8 ± 199.5		255.6 ± 334.5	
	rs11807	AA	265.5 ± 203.5	0.526	148.9 ± 91.2	0.797	166.1 ± 158.9	0.276	177.5 ± 225.7	0.731
		AG+GG	294.9 ± 222.4		175.3 ± 151.9		160.3 ± 182.5		158.5 ± 172.7	

The effects of recipient CYP3A5 and GSTM2-5 SNPs on Tac C/D ratios in the early period after LT was shown in Table 3. Tac C/D ratios of recipient CYP3A5 rs776746 AA/AG carriers were 220.7 ± 190.0, 126.2 ± 77.1, 120.5 ± 107.3 and 141.7 ± 222.3 at week 1, 2, 3 and 4 respectively, and 328.8 ± 216.7, 188.7 ± 139.9, 202.0 ± 199.6 and 195.2 ± 190.5 for GG genotype carriers. Tac C/D ratios of recipient CYP3A5 rs776746 AA/AG carriers were significantly lower than GG carriers at all investigated time points (*p* = 0.001, 0.006, 0.005, 0.003, respectively). However, there was not significant association between the recipient GSTM2-5 genotype groups in the early post-transplantation period.

**Table 3:** Tac C/D ratios according to recipient CYP3A5 and GSTMs genotypes after drug initiation (Cohort A, *n* = 110).

			Week 1		Week 2		Week 3		Week 4	
Gene	SNP	Genotype (n)	C/D ratios	P	C/D ratios	P	C/D ratios	P	C/D ratios	P
CYP3A5	rs776746	AA+AG (58)	220.7 ± 190.0	0.001	126.2 ± 77.1	0.006	120.5 ± 107.3	0.005	141.7 ± 222.3	0.003
		GG (52)	328.8 ± 216.7		188.7 ± 139.9		202.0 ± 199.6		195.2 ± 190.5	
GSTM2	rs530021	CC (93)	275.8 ± 215.3	0.729	158.9 ± 115.5	0.392	166.1 ± 168.6	0.424	174.2 ± 215.2	0.570
		CG+GG (17)	284.5 ± 175.7		159.2 ± 141.9		146.4 ± 164.1		135.1 ± 99.9	
GSTM3	rs7483	AA (59)	276.4 ± 232.5	0.519	155.4 ± 117.8	0.508	177.1 ± 190.6	0.591	177.5 ± 242.4	0.383
		AG+GG (51)	277.2 ± 183.7		163.2 ± 119.5		148.2 ± 135.5		162.2 ± 158.2	
	rs4646412	GG (101)	274.3 ± 207.4	0.855	159.4 ± 121.9	0.687	169.1 ± 177.3	0.689	175.7 ± 218.6	0.908
		GT+TT (9)	296.2 ± 241.6		155.2 ± 86.4		126.3 ± 49.3		130.8 ± 64.1	
GSTM4	rs506008	GG (96)	273.7 ± 215.1	0.419	157.9 ± 115.3	0.615	165.0 ± 168.1	0.689	174.2 ± 215.2	0.570
		GA+AA (14)	303.1 ± 171.4		167.5 ± 145.7		154.6 ± 169.6		135.1 ± 100.0	
GSTM5	rs1296954	GG (73)	303.2 ± 229.4	0.153	157.7 ± 96.9	0.429	175.9 ± 176.8	0.253	180.7 ± 231.0	0.540
		AG+AA (37)	240.8 ± 177.6		160.8 ± 145.4		146.0 ± 152.8		155.8 ± 158.1	
	rs11807	AA (67)	271.6 ± 201.8	0.916	172.2 ± 132.1	0.214	169.8 ± 177.1	0.662	159.6 ± 170.2	0.490
		AG+GG (43)	288.6 ± 231.6		130.0 ± 73.2		148.9 ± 141.6		198.3 ± 282.4	

### Multivariate analysis for factors influencing Tac metabolism (Cohort A and B)

We investigated the effect of the genetic and clinical factors on Tac metabolism in the early post-transplantation period through multiple linear regression analysis. The cofactors that were incorporated into the analysis included CYP3A5 rs776746 and donor GSTM3 rs7483, liver function indices (ALT, AST, TB, DB, Alb), and renal function indices (Cr and Urea). In the training set (Cohort A: Table 4), donor and recipient CYP3A5 rs776746, donor GSTM3 rs7483 and the liver function indices (TB, DB) were identified as independent predictors of Tac metabolism in the early period after LT.

**Table 4:** Multiple linear regression model for log-transformed Tac C/D ratios in the first month (Cohort A, *n* = 110, stepwise method).

		B	Beta	T	Sig.	VIF	Adjusted R^2^	D-W
Week1	(Constant)	1.406		8.970	.000		0.248	1.806
	Donor rs7483	.157	.225	2.574	.012	1.108		
	Donor rs776746	.225	.322	3.708	.000	1.006		
	Recipient rs776746	.232	.334	3.827	.000	1.013		
Week2	(Constant)	1.421		11.476	.000		0.268	1.897
	Total bilirubin	.001	.291	3.376	.001	1.055		
	Donor rs7483	.119	.210	2.416	.018	1.072		
	Donor rs776746	.137	.242	2.876	.005	1.005		
	Recipient rs776746	.158	.279	3.297	.001	1.018		
Week3	(Constant)	1.332		9.962	.000		0.245	2.107
	Direct bilirubin	.003	.317	3.681	.007	1.038		
	Donor rs7483	.121	.195	2.216	.029	1.053		
	Donor rs776746	.196	.317	3.681	.000	1.008		
	Recipient rs776746	.148	.239	2.772	.007	1.013		
Week4	(Constant)	1.139		8.346	.000		0.357	2.163
	Direct bilirubin	.002	.176	2.108	.038	1.047		
	Donor rs7483	.202	.323	3.884	.000	1.045		
	Donor rs776746	.279	.448	5.463	.000	1.013		
	Recipient rs776746	.142	.227	2.766	.007	1.013		

To confirm the effect of significant factors on Tac metabolism, we proceeded with further analysis in the validating set (Cohort B: Table 5). Donor and recipient CYP3A5 rs776746 and donor GSTM3 rs7483 were significantly associated with Tac elimination in the early period after LT. In addition, total bilirubin was also a predictor of Tac elimination for week 1.

**Table 5:** Multiple linear regression model for log-transformed Tac C/D ratios in the first month (Cohort B, *n* = 93, enter method).

		B	Beta	T	Sig.	VIF	Adjusted R^2^	D-W
Week1	(Constant)	1.772		9.072	.000		0.170	1.559
	Total bilirubin	.001	.249	2.231	.029	1.067		
	Donor rs7483	.108	.208	2.083	.040	1.042		
	Recipient rs776746	.194	.316	2.871	.005	1.037		
Week2	(Constant)	1.805		8.524	.000		0.084	1.173
	Donor rs776746	.219	.322	3.007	.004	1.035		
Week3	(Constant)	1.434		8.894	.000		0.207	1.743
	Donor rs7483	.140	.238	2.343	.022	1.018		
	Donor rs776746	.193	.329	3.199	.002	1.039		
	Recipient rs776746	.171	.285	2.788	.007	1.031		
Week4	(Constant)	1.410		7.583	.000		0.176	1.692
	Donor rs7483	.161	.265	2.363	.021	1.020		
	Donor rs776746	.170	.279	2.495	.015	1.015		
	Recipient rs776746	.153	.246	2.204	.031	1.014		

### Combined effects of CYP3A5 rs776746 and GSTM3 rs7483 genotypes

Donor and recipient CYP3A5 rs776746 allele A and donor GSTM3 rs7483 allele A were associated with fast Tac metabolism as stated above, we combined CYP3A5 rs776746 and GSTM3 rs7483 genotypes and investigated the effects of the number of alleles associated with fast metabolism on Tac C/D ratios (Table 6). Group 1 consisted of 0-1 alleles (poor metabolizers); group 2 contained 2-3 alleles (intermediate metabolizers); group 3 contained 4-6 alleles (extensive metabolizers). With increasing numbers of alleles associated with fast metabolism, Tac C/D ratios were increasingly lower at each time points within the first month (group 1 >group 2 >group 3; *p* = 0.001, 0.004, <0.001, <0.001, respectively).

**Table 6:** Combined analysis of donor CYP3A5 rs776746 allele A, recipient CYP3A5 rs776746 allele A and donor GSTM3 rs7483 allele A on Tac C/D ratios after drug initiation (*n* = 203).

		Num^a^			
		0–1 (Group 1, *n* = 31)	2–3 (Group 2, *n* = 131)	4–6 (Group 3, *n* = 41)	*p*-value
Month 1	Week 1	314.24 ± 259.76	224.17 ± 166.19	144.11 ± 126.04	0.001
	Week 2	268.46 ± 182.46	172.60 ± 155.42	148.13 ± 132.98	0.004
	Week 3	296.54 ± 216.00	158.52 ± 135.54	115.14 ± 70.94	<0.001
	Week 4	305.84 ± 242.48	153.84 ± 170.85	95.91 ± 70.07	<0.001

**Notes.**

a The number of alleles associated with fast metabolism.

**Figure 1:**
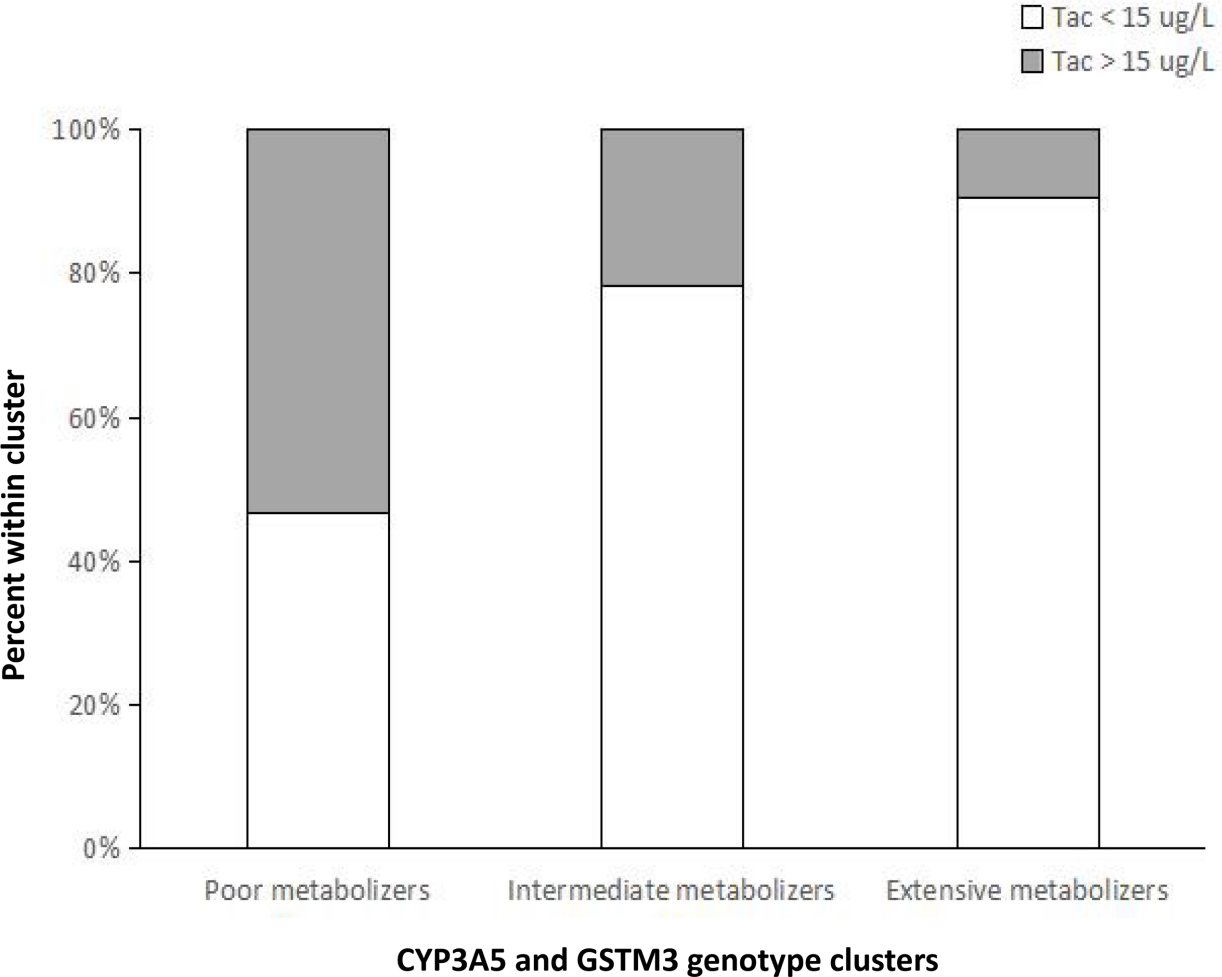
Percentage of patients within each metabolizer cluster stratified by values of C_0_ below or above the 15-ug/L supratherapeutic threshold at week 1 after liver transplantation. The risk of presenting a supratherapeutic C_0_ (Tac > 15 ug/L) at week 1 was significantly higher for poor metabolizers, compared with intermediate metabolizers ( *p* = 0.016) and extensive metabolizers (*p* = 0.008).

The geometric mean of Tac concentrations were 13.6 ug/L, 9.2 ug/L, 5.7 ug/L at week 1 for patients from group 1, group 2 and group 3, respectively. Logistic regression analysis showed that the risk of presenting a supratherapeutic C_0_ (Tac >15 ug/L) at week 1 was significantly higher for group 1 (Fig. 1), compared with group 2 (odds ratio: 4.143; 95% CI [1.305–13.175]; *p* = 0.016) and group 3 (odds ratio: 3.295 ; 95% CI [1.356–8.005]; *p* = 0.008). However, no significant differences were observed between the different groups regarding to the risk of a C_0_ <8 ug/L (*p* = 0.742, 0.163, respectively). These results indicated that poor metabolizers require lower Tac doses to reach the target blood concentrations and genotype classification demonstrated a better predictive ability for the initial Tac doses after LT.

## Discussion

The study is the first time to investigate the effects of the GSTMs genetic variants on Tac metabolism in the early period after LT. In the training set, we found that Tac C/D ratios of donor GSTM3 rs7483 AA carriers were significantly lower than those with the G allele at weeks 1, 2, 3 and 4. No significant association between the other GSTM2-5 genotype groups were observed at all investigated time points. In multiple linear regression analysis, donor GSTM3 rs7483 genetic variant was identified as an independent predictor of Tac elimination in the early period after LT both in the two cohorts. Of 203 liver transplant patients, the distribution of genotypes for the GSTM3 rs7483 genetic variant were 6.7%GG, 40.6%GA, 52.7%AA, aligning with the previous research on the Chinese population (Tan et al., 2013; Tetlow et al., 2004).

Our results are agreement with the increased function of the GSTM3 rs7483 genetic variant and the expected fast metabolism of Tac. GSTM3 belongs to the phase II drug-metabolising enzymes that plays a key role in the detoxification of chemical agents. Increasing evidences have revealed that the capacity to metabolise drugs may be partly affected by the genetic variants in the population (Liu et al., 2022; Aouam et al., 2015; Liu et al., 2013). A research had reported that the genetic variant of GSTM1 had a potential association with Tac elimination in the first month after transplantation (Singh et al., 2009). In addition, the SNP rs7483 (224 G>A) in GSTM3 results in the substitution of valine (Val) for isoleucine (Ile) in the GSTM3 protein, which has been reported to significantly increase the activity of the drug-metabolising enzyme (Tetlow et al., 2004; Shiota et al., 2016). Therefore, the increased enzymatic activity might affect Tac metabolism.

In the present study, we have further confirmed that patients with CYP3A5 rs776746 AA/AG genotype (expressers) require lower Tac doses to achieve the target blood concentration compared with CYP3A5 GG genotype carriers (nonexpressers) (Du et al., 2024a; Du et al., 2024b; Nuchjumroon et al., 2022; Dong et al., 2022; Sallustio et al., 2021; Everton Janaína et al., 2021; Kelava et al., 2020; Coller et al., 2019; Tang et al., 2019; Zhang et al., 2018). The effects of donor and recipient CYP3A5 rs776746 and donor GSTM3 rs7483 SNPs appeared independent, the combined analysis of CYP3A5 rs776746 and donor GSTM3 rs7483 genotypes shown a more significant impact on Tac pharmacokinetics compared to examining the genotypes separately. Tac C/D ratios were significantly lower with increasing numbers of alleles associated with fast metabolism: poor metabolizers (Group 1) >intermediate metabolizers (Group 2) >extensive metabolizers (Group 3). Furthermore, our results demonstrated that the risk of a supratherapeutic C_0_ (Tac >15 ug/L) at week 1 was significantly higher for poor metabolizers than for intermediate metabolizers and extensive metabolizers. Although therapeutic drug monitoring (TDM) is helpful for subsequent dosage modification, it provides no information for the initial dose. Therefore, genotype classification might help clinicians to individualize the Tac starting dose after LT.

Beyond genetic factors, clinical parameters (total bilirubin, direct bilirubin) were found to be significantly correlated with Tac elimination after LT, which was consistent with the previous study (Fan et al., 2015; Luo et al., 2016). It is well known that biliary excretion is associated with the elimination of Tac metabolites (Siqi et al., 2024), and therefore, alterations in liver function could significantly influence Tac pharmacokinetics.

There were several limitations in our study. Firstly, these results were obtained from a relatively small number of Chinese patients. Confirmation of the effects of the GSTMs SNP is need in larger or more diverse populations. Secondly, this study lack some experimental data to support the clinical observation. Therefore, further clinical and mechanistic studies are needed to elucidate our findings.

In summary, we have demonstrated that donor GSTM3 rs7483 genetic variant was associated with fast Tac metabolism in the early post-transplantation period. Combined CYP3A5 rs776746 and donor GSTM3 rs7483 genotypes could assist in the precise determination of initial Tac doses to achieve a target concentration and reduce the risk of reaching supratherapeutic concentration.

## Supplemental Information

10.7717/peerj.18360/supp-1 Table S1 Genotype frequency of CYP3A5 and GSTMs polymorphisms in liver transplant patients (Cohort A, n = 110)

10.7717/peerj.18360/supp-2 Supplemental Information 2 Drug-metabolizing enzymes and transporter (DMET) microarray The current study was based on our drug-metabolizing enzymes and transporter (DMET) microarray, which contains 1936 genetic variants in 225 related genes.

10.7717/peerj.18360/supp-3 Supplemental Information 3 The raw data of participants (Cohort A=110) In Cohort A (n=110), the raw data includes genetic variants and clinical characteristics.

10.7717/peerj.18360/supp-4 Supplemental Information 4 The Raw Data of participants (Cohort B=93) In Cohort B (n=93), the raw data includes genetic variants and clinical characteristics.

10.7717/peerj.18360/supp-5 Supplemental Information 5 Organ Donation Details (Cohort A n=110)

10.7717/peerj.18360/supp-6 Supplemental Information 6 Organ Donation Details (Cohort A n=93)

## Additional Information and Declarations

### Microarray Data Deposition

The following information was supplied regarding the deposition of microarray data:
https://www.biosino.org/node/project/detail/OEP00005561.

### Data Availability

The following information was supplied regarding data availability: The raw data are available in the Supplementary Files.
