# Supplementary material for: Effect of donor GSTM3 rs7483 genetic variant on tacrolimus elimination in the early period after liver transplantation
Source: PeerJ. 2024 Oct 23;12:e18360. doi: 10.7717/peerj.18360 (PMC11512548; doi:10.7717/peerj.18360)
Supplement: Table S1 [file peerj-12-18360-s001.docx]

Supplementary table 1. Genotype frequency of CYP3A5 and GSTMs polymorphisms in liver transplant patients (Cohort A, n = 110).

| Gene | SNP | Total population (n = 110) | | | HWE |
| --- | --- | --- | --- | --- | --- |
| CYP3A5 | rs776746 | AA | AG | GG |  |
|  | Donors | 7(0.07) | 51(0.46) | 52(0.47) | 0.23 |
|  | Recipients | 8(0.07) | 45(0.41) | 57(0.52) | 0.83 |
| GSTM1 | rs1065410 | CC | CT | TT |  |
|  | Donors | 110(1.00) | 0(0.00) | 0(0.00) | 0 |
|  | Recipients | 110(1.00) | 0(0.00) | 0(0.00) | 0 |
|  | rs737497 | AA | AG | GG |  |
|  | Donors | 110(1.00) | 0(0.00) | 0(0.00) | 0 |
|  | Recipients | 110(1.00) | 0(0.00) | 0(0.00) | 0 |
| GSTM2 | rs530021 | CC | CG | GG |  |
|  | Donors | 93(0.85) | 17(0.15) | 0(0.00) | 0.38 |
|  | Recipients | 98(0.89) | 12(0.11) | 0(0.00) | 0.55 |
|  | rs592792 | CC | CT | TT |  |
|  | Donors | 109(0.99) | 1(0.01) | 0(0.00) | 0.96 |
|  | Recipients | 110(1.00) | 0(0.00) | 0(0.00) | 0 |
|  | rs1056799 | TT | TA | AA |  |
|  | Donors | 110(1.00) | 0(0.00) | 0(0.00) | 0 |
|  | Recipients | 110(1.00) | 0(0.00) | 0(0.00) | 0 |
|  | rs1056800 | GG | GA | AA |  |
|  | Donors | 110(1.00) | 0(0.00) | 0(0.00) | 0 |
|  | Recipients | 110(1.00) | 0(0.00) | 0(0.00) | 0 |
|  | rs11540636 | TT | TC | CC |  |
|  | Donors | 110(1.00) | 0(0.00) | 0(0.00) | 0 |
|  | Recipients | 110(1.00) | 0(0.00) | 0(0.00) | 0 |
| GSTM3 | rs7483 | GG | GA | AA |  |
|  | Donors | 9(0.08) | 42(0.38) | 59(0.54) | 0.69 |
|  | Recipients | 8(0.07) | 44(0.40) | 58(0.53) | 0.93 |
|  | rs4646412 | GG | GT | TT |  |
|  | Donors | 101(0.92) | 9(0.08) | 0(0.00) | 0.65 |
|  | Recipients | 97(0.88) | 13(0.12) | 0(0.00) | 0.51 |
|  | rs2234696 | AA | AC | CC |  |
|  | Donors | 110(1.00) | 0(0.00) | 0(0.00) | 0 |
|  | Recipients | 110(1.00) | 0(0.00) | 0(0.00) | 0 |
|  | rs1803688 | GG | GT | TT |  |
|  | Donors | 110(1.00) | 0(0.00) | 0(0.00) | 0 |
|  | Recipients | 110(1.00) | 0(0.00) | 0(0.00) | 0 |
|  | rs1803687 | GG | GC | CC |  |
|  | Donors | 110(1.00) | 0(0.00) | 0(0.00) | 0 |
|  | Recipients | 110(1.00) | 0(0.00) | 0(0.00) | 0 |
|  | rs67174253 | AA | AC | CC |  |
|  | Donors | 110(1.00) | 0(0.00) | 0(0.00) | 0 |
|  | Recipients | 110(1.00) | 0(0.00) | 0(0.00) | 0 |
| GSTM4 | rs506008 | GG | GA | AA |  |
|  | Donors | 96(0.87) | 14(0.13) | 0(0.00) | 0.48 |
|  | Recipients | 99()0.90 | 11(0.10) | 0(0.00) | 0.58 |
|  | rs3211193 | CC | CA | AA |  |
|  | Donors | 110(1.00) | 0(0.00) | 0(0.00) | 0 |
|  | Recipients | 110(1.00) | 0(0.00) | 0(0.00) | 0 |
| GSTM5 | rs1296954 | GG | GA | AA |  |
|  | Donors | 73(0.66) | 35(0.32) | 2(0.02) | 0.34 |
|  | Recipients | 64(0.58) | 44(0.40) | 2(0.02) | 0.07 |
|  | rs11807 | AA | AG | GG |  |
|  | Donors | 67(0.61) | 40(0.36) | 3(0.03) | 0.30 |
|  | Recipients | 77(0.70) | 31(0.28) | 2(0.02) | 0.58 |
|  | rs2479390 | AA | AG | GG |  |
|  | Donors | 110(1.00) | 0(0.00) | 0(0.00) | 0 |
|  | Recipients | 110(1.00) | 0(0.00) | 0(0.00) | 0 |
|  | rs2229059 | TT | TC | CC |  |
|  | Donors | 110(1.00) | 0(0.00) | 0(0.00) | 0 |
|  | Recipients | 110(1.00) | 0(0.00) | 0(0.00) | 0 |
|  | rs1049741 | GG | GC | CC |  |
|  | Donors | 110(1.00) | 0(0.00) | 0(0.00) | 0 |
|  | Recipients | 110(1.00) | 0(0.00) | 0(0.00) | 0 |
